# The acute management of Pilon fractures (ENFORCE) study: a national evaluation of practice

**DOI:** 10.1308/rcsann.2024.0063

**Published:** 2024-12-13

**Authors:** DS Hill, JR Davis, ENFORCE Collaborative

**Affiliations:** Torbay and South Devon NHS Foundation Trust, UK

**Keywords:** Management, Pilon fractures

## Abstract

**Introduction:**

Pilon fractures are potentially limb-threating injuries. Staged soft tissue damage control is described, but actual practices are unknown. We report a national trainee collaborative evaluating how tibial Pilon fractures are acutely managed.

**Methods:**

ENFORCE was a multicentre retrospective observational study of the acute management of tibial Pilon fractures over a three-year period. Mechanism of injury, imaging, fracture classification, time to cast application, soft tissue damage control strategy and definitive management details were determined.

**Results:**

A total of 656 patients (670 fractures) across 27 centres were reported. AO fracture classifications were: partial articular (*n*=294) and complete articular (*n*=376). Initial diagnostic imaging mobilities were plain radiographs (*n*=602) and a trauma computed tomography (CT) scan (*n*=54). A total of 526 fractures had a cast applied in the emergency department (91 before radiological diagnosis), with the times taken to obtain postcast imaging being: mean 2.7 hours, median 2.3 hours and range 28 minutes–14 hours. 35% (102/294) of partial articular and 57% (216/376) of complete articular fractures had a spanning external fixator applied, all of which underwent a planning CT scan. Definitive management consisted of open reduction internal fixation (*n*=495), fine wire frame (*n*=86), spanning external fixator (*n*=25), intramedullary nail (*n*=25) or other (*n*=18).

**Conclusion:**

The management of tibial Pilon fractures is variable, with just over half of complete articular fractures managed with the gold standard ‘span, scan, plan’ staged soft tissue resuscitation. A national standard of care would increase the profile and standardise management of these potentially limb-threatening injuries, together with setting them apart from more straightforward ankle fractures.

## Introduction

Tibial Pilon fractures are severe injuries of the articular surface of the tibial plafond. They are reported to represent around 1% of all lower limb fractures,^1^ but the true incidence is unknown.^2,3^ Uncertainty exists around which fracture patterns should be considered ‘Pilon’ fractures, and how they should be managed.^4^ The ideal classification system does not exist. The AO classification considers distal tibial fractures as being extra-articular, partial articular, or complete articular.^5^ Ruedi^6^ and Topliss^7^ have also described classification systems, but none have described the extent of soft tissue damage. Factors such as fracture blisters, soft tissue injury or compromise are important features but are problematic to quantify.^8^ Best outcomes are achieved with anatomical reconstruction, with fracture union, in the absence of wound breakdown and resultant infection.^7^ Problems with delayed union and infection are common, and functional outcomes are often poor.^9^ The management strategy employed is dependent on the severity of bony and soft tissue injuries, patient factors, surgeon training, local expertise, resources and experience.

Understanding the pathophysiology of the soft tissue trauma and management options is of paramount importance for surgeons treating these injuries. Failure to fully appreciate this has the potential to cause dramatic complications such as soft tissue loss, prolonged treatment time, infection, multiple surgeries and even amputation.^1^ Strategies for controlling skeletal alignment and achieving soft tissue resuscitation include a plaster cast, a spanning external fixator and early definitive surgical management. Early fixation (on the day or day after injury) is recommended in the context of ankle fractures where the mortice is unstable^10^; however, no guidance exists advocating this strategy for distal tibial Pilon fractures. Some well-resourced units can offer early open reduction and internal fixation with soft tissue reconstruction if required. This is not universally available and risks infection, requirement for plastic surgical reconstruction and amputation.^11^

A staged approach to achieve soft tissue resuscitation with temporary external fixation then obtaining cross-sectional imaging to aid operative planning (‘Span, Scan, Plan’) has been popularised.^11,12^ Maintaining the tibia out to length and disimpacting the talus from the tibial fragments, preventing further joint surface damage and countering soft tissue contracture, is particularly important in length-unstable injuries where it is not possible to maintain the talus out to length under the tibial plafond with cast immobilisation alone. The limited soft tissue coverage around the ankle means the adjacent soft tissues are particularly susceptible to compromise. This predisposes to both the effects of the index primary traumatic event on the soft tissue envelope, and also the delayed secondary swelling from ongoing skeletal instability/deformity if the fracture is not grossly reduced and held out to length. Part of the inflammatory cascade causes capillary system endothelial permeability, which can incite uncontrolled swelling.^8^

Successful management of the soft tissues is of paramount importance for a good outcome following a tibial Pilon fracture, and failure to adequately achieve soft tissue resuscitation at an early stage may contribute, in part, to the poor outcomes reported following a tibial Pilon fracture. An ankle fracture BOAST (British Orthopaedic Association Standards for Trauma) endorsed by the British Orthopaedic Foot and Ankle Society (BOFAS) was published in 2016,^10^ but the same has not been produced for tibial Pilon fractures. Given the complexity of managing tibial Pilon fractures, the high incidence of complications and associated costs of managing their complications, the need for subspeciality management guidance is clear.

The primary aim of this study was to evaluate practices around acute soft tissue damage control management in patients with a tibial Pilon fracture. The secondary aims included determining the utilisation of a staged approach to achieve soft tissue resuscitation with temporary external fixation and obtaining cross-sectional imaging to aid in operative planning (‘Span, Scan, Plan’), and to determine how these injuries were definitively managed. We did not aim to comment on superiority of different acute or definitive management strategies.

## Methods

### Design

The study protocol was trialled at a regional Major Trauma Centre (University Hospitals Plymouth). Pilot data consisted of 12 patients (with 13 Pilon fractures) retrospectively identified during a 12-month period (Supplemental file provided.) Scientific review of the study protocol was provided with kind support by the BOFAS scientific committee.

### Site registration and teams

Trauma and Orthopaedic Surgery Higher Training regions with collaborative research groups are listed on the British Orthopaedic Trainees Association website. Each was contacted and invited to advertise the ENFORCE study in their region. An overview of the study was provided together with details of how to register and participate. This advertisement was also distributed to the national training programme directors forum, with a request to disseminate to all trainees. We specified that each locality team should consist of at least one consultant orthopaedic surgeon with a subspeciality practice in foot and ankle surgery, one ST3–ST8 higher surgical trainee, plus (or not) one other trainee. We required a consultant foot and ankle specialist to ensure the accuracy of adherence to our inclusion criteria (i.e., they considered cases submitted to be Pilon fractures) and to undertake the fracture classifications. This national service evaluation was badged as the ENFORCE study and consisted of 27 centres (Table 1), with 79 investigators (27 consultant specialist foot and ankle consultant surgeons, and 52 trainees).

**Table 1:** Trauma and Orthopaedic Units submitting patients to the ENFORCE study

Addenbrooks Hospital, Cambridge
Altnagelvin Area Hospital, Londonderry
Basingstoke and North Hampshire Hospital
Bristol Royal Infirmary
Cumberland Infirmary, Carlisle
John Radcliffe Hospital, Oxford
Lincoln County Hospital
Liverpool Royal Infirmary
Musgrove Park Hospital
North Devon District Hospital
Northampton General Hospital
Pilgrim Hospital, Boston
Poole General Hospital
Princess Royal Hospital, Telford
Queen Elizabeth University Hospital, Glasgow
Royal Berkshire Hospital
Royal Devon and Exeter Hospital
Royal Oldham Hospital
Royal United Hospital
South Devon Foundation Health Care Trust
St George's Hospital
St Richard's Hospital, Chichester
Stoke Mandeville Hospital
The Royal Cornwall Hospital
University Hospital Coventry
University Hospitals Plymouth

### Inclusion and exclusion criteria

The protocol specified that patients should be identified retrospectively over a three-year period (1 January 2020–31 December 2022). Due to the diverse nature of electronic systems in units, we did not stipulate how this should be achieved. We provided details of how case identification was undertaken in the pilot study that included searching electronic theatre systems for all operative cases coded as ‘ankle fractures’, ‘distal tibial fractures’ and ‘Pilon fractures’. The locality consultant collaborator was requested to advise on the best methods for identifying cases in their unit.

Inclusion criteria were specified as patients with complete articular (AO 43C) and partial articular (AO 43B) tibial Pilon fractures, presenting within 24 hours of injury, that required intervention in the operating theatre of any type. Investigators could find it challenging to radiologically differentiate a posterior malleolus ‘ankle fracture’ sustained from a rotational mechanism from a posterior tibial ‘Pilon fracture’. Our protocol provided an extract from Mason *et al*^13^ to provide guidance (Supplemental file provided). We also stipulated that the consultant collaborator with a subspeciality practice in foot and ankle surgery must confirm that they considered cases contributed to the ENFORCE study to be ‘Pilon’ fractures.

Exclusion criteria included: patients under the age of 18 years, patients presenting greater than 24 hours after index trauma and patients with presumed pathological processes (e.g., atraumatic injuries/Charcot-type processes).

### Data collection and management

No patient identifiable information was collected, uploaded or stored. All data were collected and managed through a secure server based at the Royal Devon University Hospitals NHS trust running the Research Electronic Data Capture (REDCap) web application. This is a software solution secure web application designed for building and managing secure online data-capture tools and databases to support clinical research.^14^ To optimise data validity and security each collaborator had their own login, which required two-factor identification. Collaborators were allocated to a specific data access group (i.e., their hospital) and could view data only from their unit. Details were entered using predefined datapoints on a template (Supplemental file provided). Demographics included: age, mechanism of injury and whether the patient was multiply injured. Fracture-specific details included laterality, primary radiological diagnosis modality (including date and time), whether a plaster cast had been applied at any point in the emergency department, and whether imaging was obtained after application of a plaster cast (including date and time). Fractures were classified according to AO^5^ and Ruedi^6^ by our consultant collaborators. Details of intraoperative Gustillo–Anderson classifications for open fractures were sought.^15^ Application of a spanning external fixator, if a computed tomography (CT)-scan was performed and when, and how the fracture was definitively managed, were also collected.

### Ethical approval

Each participating unit was required to obtain local clinical governance approval before data collection. The Health Research Authority (HRA) decision tool was undertaken and confirmed that this study would not require an application and approval by a Research Ethics Committee.

## Results

### Patients and demographics

We present data for 656 patients (with 670 fractures) who underwent intervention in the operating theatre of any kind for a tibial Pilon fracture during a three-year period at 27 centres (9 major trauma centres and 18 regional trauma units); 60% (*n*=399) of patients were male and 40% (*n*=257) were female. Mean age at time of injury was 47 years (median 47 years, range 18–87 years). Mechanisms of injury were fall <2m (*n*=325), fall >2m (*n*=240), road traffic collision (*n*=81), crush injury (*n*=5) and industrial accident (*n*=5). A total of 118 patients were reported as being multiply injured, with 7 having bilateral Pilon fractures.

### Emergency department

**Figure 1:**
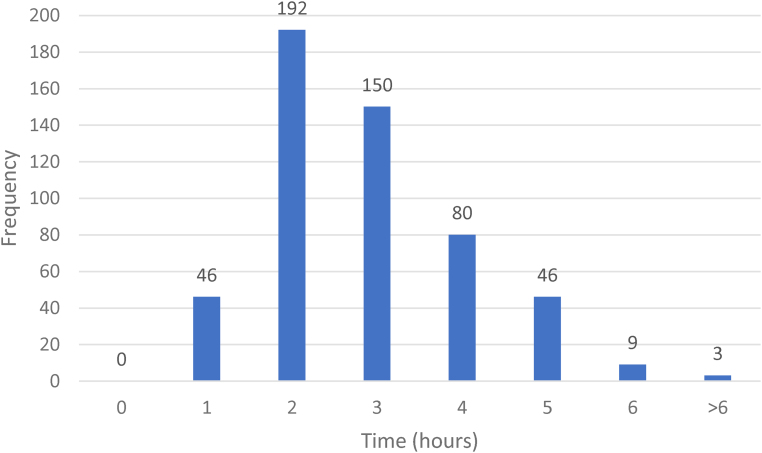
Distribution of time between primary radiological diagnosis and subsequent imaging following application of a plaster cast in the emergency department

Initial diagnostic imaging mobilities were as follows: 92% (*n*=602) plain radiographs and 8% (*n*=54) a trauma CT-scan where whole body scanning was performed. A total of 79% (*n*=526/670) of fractures had a plaster cast applied in the emergency department (91 before primary radiological diagnosis, implying a significant initial displacement). Following initial diagnostic imaging, 21% (*n*=144/670) were transferred directly to the operating theatre for emergency intervention without plaster cast application, of which 94 were open fractures (Table 2). Where a plaster cast was applied in the emergency department, the times taken from primary radiological diagnosis to postcast imaging being performed were as follows: mean 2.7 hours, median 2.3 hours, range 28 minutes–14 hours (Figure 1).

**Table 2:** Classification and acute management of distal tibial Pilon fractures*

	AO Group B 44% (*n*=294)			AO Group C 56% (*n*=376)		
Ruedi classification	Ruedi Type 1	Ruedi Type 2	Ruedi Type 3	Ruedi Type 1	Ruedi Type 2	Ruedi Type 3
	24% (71/294)	46% (136/294)	30% (87/294)	6% (23/376)	27% (103/376)	67% (250/376)
Primary soft tissue damage control	Plaster cast (*n*=71)	Plaster cast (*n*=136)	Plaster cast (*n*=87)	Plaster cast (*n*=23)	Plaster cast (*n*=91)	Plaster cast (*n*=118)
					Direct transfer to theatre (*n*=34)	Direct transfer to theatre(*n*=110)
Spanning external fixator applied at any point	24% (17/71)	29% (40/136)	52% (45/87)	30% (7/23)	83% (85/103)	82% (206/250)
Time from primary radiological diagnosis to application of spanning external fixator	Mean: 36.6 hours	Mean: 31.2 hours	Mean: 41.9 hours	Mean: 16.0 hours	Mean: 35.3 hours	Mean: 35.2 hours
	Median: 20.2 hours	Median: 19.5 hours	Median: 19.2 hours	Median: 14.4 hours	Median: 19.7 hours	Median:19.1 hours
	Range: 5.8 hours–5 days	Range: 4.0 hours–8 days	Range: 1.9 hours–13.2 days	Range: 4.0 hours–23 hours	Range: 3.8 hours–17 days	Range: 1.8 hours–12 days
Span, Scan, Plan	24% (17/71)	29% (40/136)	52% (45/87)	30% (7/23)	83% (85/103)	82% (206/250)

*AO Group B: Partial articular injury; AO Group C: Complete articular injury; Ruedi Type 1: No comminution or displacement of joint fragments; Ruedi Type 2: Some displacement, but no comminution or impaction; Ruedi Type 3: Comminution and/or impaction of joint surface

### Fracture classifications

A total of 81 Pilon fractures were reported as open fractures at presentation, with subsequent intraoperative Gustillo–Anderson classifications of grade 1 (*n*=14), grade 2 (*n*=17), grade 3A (*n*=10), grade 3B (*n*=28) or grade 3C (*n*=1). In 11 patients who had an open fracture, no classification was recorded (Table 2).

### Span, scan, plan

A total of 35% (102/294) of AO Type B partial articular and 57% (216/376) of AO Type C complete articular (length-unstable) fractures had an external fixator applied at any point in their acute management, all of which underwent a planning CT scan. Durations from primary radiological diagnosis to application of an external fixator for all fractures were as follows: mean 35 hours, median 19.2 hours and a range of 1.8 hours–10 days. Increasing severity of comminution and joint surface impaction as indicated by reported Ruedi classifications were associated with increased use of a spanning external fixator. A total of 96% (631/656) of fractures underwent a CT scan before definitive management; however, 48% (318/656) underwent staged soft tissue resuscitation management in an external fixator followed by a subsequent planning CT scan (Table 2). The remaining 313 fractures underwent a CT-scan before the application of a spanning external fixator.

### Major trauma centre versus regional trauma unit

In our series, 49% (277/656) of patients presented initially to a major trauma centre and 51% (339/565) to regional trauma units. A total of 376 fractures were classified as AO type C complete articular (length unstable), of which 79% (298/376) had a spanning external fixator placed. A comparison of time from primary radiological diagnosis to application of a spanning external fixator in the major trauma centres versus the regional trauma units is presented in Table 3.

**Table 3:** Time from primary radiological diagnosis to application of a spanning external fixator in patients with an AO Type C complete articular (length-unstable) distal tibial Pilon fracture where a spanning external fixator was placed to achieve soft tissue resuscitation

	Major trauma centre	Regional trauma unit
Time from primary radiological diagnosis to application of spanning external fixator	Mean: 19.1 hours	Mean: 19.2 hours
	Median: 16.4 hours	Median: 18.6 hours
	Range: 2.7 hours–2.8 days	Range: 2.6 hours–2.2 days

### Definitive management

Details of definitive management were reported for 97% (648/670) of fractures. This consisted of open reduction internal fixation 74% (*n*=481), 13% fine wire frame (*n*=87), intramedullary nail 4% (*n*=27), spanning external fixator 4% (*n*=25), close contact cast immobilisation applied in the operating theatre 4% (*n*=23) and primary amputation 1% (*n*=5) (Table 4). A total of 21 patients had a spanning external fixator applied, but were transferred to other units for definitive management, and 1 patient died with an external fixator in situ. Of the 81 open fractures: 5 were managed with primary amputation during first operative visit, and 32 required plastic surgical soft tissue coverage at the time of definitive fracture fixation.

**Table 4:** Definitive management of patients with distal tibial Pilon fractures

AO Group B 44% (*n*=294)	AO Group C 56% (*n*=376)
Open reduction internal fixation (*n*=248) Fine wire frame (*n*=13) Intramedullary nail (*n*=8) Spanning external fixator (*n*=7) Cast immobilisation (*n*=14)	Open reduction internal fixation (*n*=233) Fine wire frame (*n*=74) Intramedullary nail (*n*=19) Spanning external fixator (*n*=18) Cast immobilisation (*n*=9) Amputation (*n*=5)
Other Patient repatriated to local hospital with spanning external fixator in situ (*n*=4)	Other Patient died with spanning external fixator in situ (*n*=1) Patient repatriated to local hospital with spanning external fixator in situ (*n*=17)

## Discussion

We report a large series across a spectrum of regions, hospitals, emergency departments, on-call orthopaedic teams, and foot and ankle specialists. Reported patient age, gender and mechanisms of injury were comparable with previously published studies.^1,11,16^ Mechanisms of injury were reported in all patients, with 84% of fractures (565/670) relating to a fall of some description. Although instructional articles exist around the management^1,11,16–18^ and outcomes following tibial Pilon fracture have been published,^6,9,12,19,20^ we are not aware of any published series comparable with our own reporting on actual practices in the acute phase management of these injuries.

### Initial soft tissue damage control

We report that 79% (526/670) of fractures had a plaster cast applied in the emergency department, with a median time of 2.3 hours from primary radiological diagnosis to postplaster cast imaging being obtained, although many waited up to 4 hours (Figure 1). This is an underestimation of the true time many would have been clinically deformed and the soft tissues compromised. We are unable to account for prehospital time and delays in obtaining initial imaging, and for this to be reviewed and acted upon. Furthermore, we are unable to comment on the adequacy of joint reductions. There are no series comparable with our own reporting on time from initial radiological diagnosis to follow-up postcast imaging.

### Span, scan, plan

The concept of using a temporary spanning external fixator across the ankle joint to provide bony stability and resuscitate the soft tissues has been reported and has gained popularity.^12,21^ Numerous constructs have been proposed but no consensus exists.^18,22^ Although this strategy has been shown to be superior to plaster cast immobilisation,^23,24^ there is no Level 1 evidence to support its use. In some well-resourced trauma centres with readily available appropriate orthopaedic and plastic surgery services, an argument exists for performing the definitive surgical management in the first 24 hours, with early soft tissue reconstruction if required. This is, however, not possible universally, and no Level 1 evidence exists to support this strategy over a staged soft tissues resuscitation in a spanning external fixator before definitive management.

We found that a third of AO type B partial articular fractures (potentially length unstable) and two-thirds of AO type C complete articular fractures (length unstable) had a spanning external fixator applied to control soft tissue resuscitation, with a median time of 19.2 hours from primary radiological diagnosis to application of external fixator. During this time, it is likely that continued uncontrolled swelling will have occurred with ongoing damage to the soft tissues at both a macroscopic and microscopic level.^8^ Analysis of the most severe injuries (AO type C complete articular fractures) showed a similar median timeframe, with no difference between major trauma centres and regional trauma units (Table 3). Our experience has been that emergency departments, emergency theatre teams and anaesthetists, and indeed some orthopaedic surgeons do not differentiate between the more common ankle fractures and less common tibial Pilon fractures when it comes to assigning urgency with which to intervene to achieve joint reduction and soft tissue damage control. In part this stems from a failure to recognise a length-unstable complete articular tibial Pilon fracture as a potentially limb-threating injury, but also reflects overstretched services and a lack of formal national standards of care.

The observed variation in use of spanning external fixator is explained, in part, by severity of joint surface damage; however, 18% of the most severe injuries (Complete articular, Ruedi Type 3) were managed without the use of a spanning external fixator to control the soft tissues. This could be explained by varying orthopaedic opinions and a lack of national standards of care. Almost all fractures in our series underwent a CT scan, although around half received this before the application of a spanning external fixator. There are no similar published studies for comparison. We suggest that a combination of increased availability of whole-body trauma CT scanning in major trauma centres and increased availability of CT scanning before patients leave the emergency department in comparison with when the mantra ‘span, scan, plan’ was initially proposed could explain this.^12,21^

### Definitive management

Nonoperative management in a cast is reserved for minimally displaced fractures in patients with low physical demands who carry high risks for surgery. Multiple strategies are described for surgical management of Pilon fractures, but no Level 1 evidence exists. Options include formal open reduction and internal fixation, and external fixation with or without limited open reduction and internal fixation. In our heterogeneous series, three-quarters of fractures were managed definitively with open reduction internal fixation, with a much smaller proportion being managed with a fine wire frame ± limited open reduction internal fixation. The ACTIVE trial^3^ is currently evaluating whether internal fixation or external fixation with a fine wire frame are equivalent, superior or inferior management strategies for complete articular tibial Pilon fractures. The group aims to compare patient-reported outcome measures, infection, further unplanned surgery and cost. However, this may not be generalisable to units where the expertise to manage such injuries with a fine wire frame is lacking, and indeed whether the outcome of requirement for further surgery to address ankle joint arthritis will be affected.

### Standards of care

Since 2008, the British Orthopaedic Association has published a number of subspeciality-endorsed Standards for Trauma (BOAST) that aim to standardise care. A standard of care for tibial Pilon fractures is mandated to increase the profile and standardise acute management of these potentially limb-threatening injuries, together with setting them apart from more straightforward ankle fractures. Given the heterogeneity of patients, mechanisms of injury and resultant damage to soft tissues and bone, a clear panacea to guide the management of these complex injuries is a long way off. We have proposed standards of care supported by a large group of consultant orthopaedic surgeons for the acute soft tissue damage control, which could contribute towards a BOAST for the management of tibial Pilon fractures.^4^

### Proposed standard of care for the acute management of tibial Pilon fractures

- The first line management of a tibial Pilon fracture is to achieve soft tissue damage control.
- In length stable injuries this should be achieved with a closed reduction and application of a plaster cast, with the ankle joint remaining reduced and radiologically documented within 6 hours of primary radiological diagnosis.
- In length unstable injuries this should be achieved with spanning external fixator within 6 hours of primary radiological diagnosis.
- Joint reduction must be radiographically confirmed before transfer from the Emergency Department. If this is not possible then the patient should be transferred directly to the operating theatre for intervention to achieve soft tissue damage control. Provision for this must exist 24 hours a day.
- Intervention to achieve soft tissue damage control in a tibial Pilon fracture is an indication for rapid sequence induction anaesthesia if a patient is not appropriately starved.

### Limitations

This series represents a sample of the total population of patients with distal tibial Pilon fractures during the study period. Our rationale for only including patients managed at any point in the operating theatre was to provide a standardised approach for case identification. However, this will have excluded patients not managed in the theatre. In addition, patients presenting to a regional trauma unit emergency department and transferred directly to a major trauma centre may have been missed. Ultimately, it is unclear why the early management of tibial Pilon fractures was variable in our series. Due to its retrospective nature, our collaborators are reporting upon prerecorded datapoints. Consequently, the data presented may not be entirely accurate. Even among experts the defining features of a tibial Pilon fracture can be ambiguous, together with their radiological classification. However, by having a consultant orthopaedic surgeon with a subspeciality interest in foot and ankle surgery taking responsibility for ensuring the accuracy and validity of both we hope to have optimised the quality of cases included in this series. In addition, accurately determining the points in time at which events occurred retrospectively can be difficult. Through utilising dates and times recorded on radiological episodes (e.g., primary radiological diagnosis, postcast x-ray, and theatre image intensifier imaging), we have relative certainty in these datapoints. We are, however, unable to report upon datapoints including actual time of injury, time of presentation to hospital and time of joint reduction and cast application. No data around outcomes or complications were collected.

## Conclusion

In our series, the initial emergency management parameters of tibial Pilon fractures were variable, including prolonged delays in obtaining postcast reduction radiographs, and the indication and timing of application of an external fixator. Just over half of length-unstable complete articular fractures were managed with the gold standard ‘span, scan, plan’ staged soft tissue resuscitation, implying a failure to differentiate a tibial ‘Pilon’ fracture from the more common ‘ankle’ fracture. A BOFAS subspeciality BOAST for the ‘acute management of tibial Pilon fractures’ would aid in the standardisation of the acute management of these potentially limb-threatening injuries and increase their profile in acute care settings and orthopaedic departments, while clearly setting them apart from more straightforward ankle fractures.
